# The propensity of invasive circulating tumor cells (iCTCs) in metastatic progression and therapeutic responsiveness

**DOI:** 10.1002/cam4.2218

**Published:** 2019-05-21

**Authors:** Huan Dong, Shaun Tulley, Qiang Zhao, Leong Cho, Donghai Chen, Michael L. Pearl, Wen‐Tien Chen

**Affiliations:** ^1^ Stony Brook Medicine Stony Brook New York; ^2^ Vitatex Inc Stony Brook New York

**Keywords:** iCTCs, metastasis, ovarian cancer, therapy response, tumor invasion

## Abstract

Circulating tumor cells (CTCs) are important clinical indicators of metastatic progression and treatment efficacy. However, because of their low number and heterogeneity, reliable patient‐derived CTC models are not readily available. We report here the isolation and characterization of the invasive population of CTCs, iCTCs, from blood of 10 patients with epithelial ovarian cancer (EOC) and one pancreatic cancer patient based on the avidity of tumor cells toward an artificial collagen‐based adhesion matrix (CAM), in comparison with tumor progenitor (TP) cells isolated from tumor cell lines, tumors and ascites from EOC patients. CAM‐avid cells identified to be iCTCs were indistinguishable with TP cells using either functional CAM uptake or surface markers (seprase and CD44). In addition, iCTCs were characterized using peritoneal and spontaneous metastasis models in vivo to evaluate their metastatic propensity and therapeutic response. TP cells and iCTCs had a doubling time of about 34‐42 hours. TP cells were rare (<3.5%) in most patient‐derived specimens, however, iCTCs emigrated into blood, at a high frequency, 64.2% (n = 49). Approximately 500 patient‐derived iCTCs recapitulated formation of iCTCs in mouse blood and formed micrometastases in the liver and/or lung, a degree of metastatic spread equivalent to the inoculation of 5 × 10^5^ bulk tumor cells isolated from ascites and tumors. iCTCs were shown to be novel therapeutic targets for blocking metastasis using the reduced formation of iCTCs and micrometastases by RNAi, peptides, and monoclonal antibodies against seprase.

## 1 INTRODUCTION

Metastasis is the major cause of cancer death. However, the population of tumor cells that generates metastasis remain far less understood. It is believed that the metastasis process is driven by cancer stem‐like cell or tumor progenitor (TP) cells in tumors, followed by active spreading of TP cells through the bloodstream, as circulating tumor cells (CTCs), to distant organs.^1^, ^2^, ^3^ Despite this classic assumption, several lines of recent evidence strongly suggest that CTCs contain a less‐well defined cellular population that has potential as a clinical indicator for metastatic progression and therapeutic response in patients with nonhematopoietic cancer. Xenograft assays have demonstrated that CTCs could generate metastasis in mice,^4^, ^5^ suggesting that enumeration of a subpopulation of CTCs determined by a given CTC enrichment method could be an important clinical predictor for metastatic progression of cancer patients. In addition, CTCs provide a source of readily available and sequentially sampled invasive cancer cells suitable for developing ex vivo cultures as patient‐specific tumor models for the individualized therapeutic evaluation of cancers.^4^, ^6^, ^7^ However, the efficiency in obtaining CTC cultures using these methods is low (<20%), except for a short‐term CTC cluster method showing efficiency of up to 60%.^6^ Importantly, CTC lines were developed using blood samples from a patient with metastatic colorectal cancer collected before and after chemotherapy and targeted therapy, and during cancer progression to show changes in mRNA and protein expression (eg, DEFA6, ABCB1, and GAL) over time of treatment.^8^ This paper shows that CTCs retain common genetic traits during therapy but may develop treatment‐resistant phenotypic characteristics over time. Consistent with the observation that CTCs are heterogeneous in nature and that a specific CTC population isolated by a given method is a predictor for therapeutic response, SMAD4 expression of a CTC population called circulating tumor and invasive cells (CTICs) was able to predict response of pancreatic ductal adenocarcinoma.^9^ In addition, CTCs expressing fibroblast activation protein alpha (FAPα)/seprase and epithelial cell adhesion molecule are distinct subpopulations of CTCs and the use of these markers in concert could provide information concerning therapeutic response.^10^

To address the low number and heterogeneity of CTCs under investigation, here we used the functional collagen‐based adhesion matrix (CAM) enrichment method capable of enriching both viable bulk tumor cells and TP cells in various cellular sources from patients with high sensitivity^11^ to develop a novel patient‐derived CTC model for predicting metastatic progression and treatment response. We identified TP cells by their invasive phenotype including expression of functional CAM uptake and markers for epithelial stem cells such as CD44 and invasiveness (seprase), the method identical to that identifying iCTCs in blood of patients with both metastatic and early stage cancers of the breast, ovary, gastrointestines, lung, pancreas, and prostate.^1^, ^11^, ^12^, ^13^, ^14^, ^15^, ^16^, ^17^, ^18^, ^19^ We demonstrated potential roles of patient‐derived iCTCs in metastatic progression and therapeutic response in experimental mice using appearance of mouse iCTCs and micrometastases in the lung or liver as well as after inoculating experimental mice with cells with altered gene expression via RNAi, peptides, and monoclonal antibodies respectively, against seprase.

## 2 MATERIALS AND METHODS

### 2.1 Patients, blood, ascites and tissue collection, and tumor cell lines

The primary objective is to characterize TP cells in tumor tissue, ascites, and blood from EOC patients. This study was approved by the institutional review board overseeing human research at Stony Brook University. Female patients at least 18 years of age were included. Patients consented to participate in this study including collection of blood and otherwise discarded ascites and tissue specimens and their clinical status and treatment plan without their personal information.

Blood collection and transport were previously described.^14^ Briefly, 2‐20 mL of blood was collected from patients using Vacutainer® tubes (Becton Dickinson; green top, sodium heparin as anticoagulant) and processed within 48 hours from collection. Blood was kept at 2‐8°C when storage longer than 4 hours was needed.

LOX human malignant melanoma line was obtained as described 20 and MDA‐MB‐231 human breast carcinoma and other tumor cell lines used in this study were obtained from American Type Culture Collection (Manassas, VA). Choice of LOX cells in this study is that the tumor cell line is the most reliable line to generate up to 25% of the cellular population that are positive in the CAM invasion assay and the spontaneous metastasis mouse model used in this study.

### 2.2 Characterization of TP and bulk tumor cells in vitro

To characterize TP and bulk tumor cells, we used CAM‐coated Vita‐Assay™ plates (Vitatex Inc, Stony Brook, NY) for the prospective isolation of CAM‐avid tumor cells from LOX human malignant melanoma^20^ and MDA‐MB‐231 human breast carcinoma and other cell lines (American Type Culture Collection, Manassas, VA), as well as from primary/metastatic tumors, ascites and blood from 49 EOC patients. Aliquots of CAM‐avid cells were left in the plate for 1‐3 days to enhance signal of cellular functions, that is, CAM uptake. Numbers of TP and bulk tumor cells were counted manually by microscopy or by automated flow cytometry according to methods previously described.^11^

### 2.3 Spontaneous and peritoneal metastasis models

All animal experiments were approved by the Stony Brook University Institutional Animal Care and Use Committee. To examine the metastatic propensities of TP cells, specimens obtained from solid tissue at primary and metastatic sites, ascites and blood of 14 ovarian cancer patients (designated P1‐P14, Table 1) and blood of a pancreatic cancer patient (designated P15, Table 1) were used to isolate viable Epi^+^ and CAM^+^tumor cells by CAM‐coated plates for subsequent injection into FOX Chase SCID mice (Taconic, Hudson, NY) either *sc* (subcutaneous injection, the spontaneous metastasis model) or *ip* (Intraperitoneal injection, the peritoneal metastasis model). Tumor cells derived from blood and solid tissue of cancer patients were injected *sc* into flanks of mice according to the procedure of the spontaneous metastasis model previously described.^20^ Tumor cells derived from ascites were injected *ip* for the peritoneal metastasis model, as previously described.^21^ The capability of cells injected *ip* or *sc* to proliferate at the injection site and perhaps forming palpable tumors was used as a measure of tumor growth. Appearance of tumor cell clusters in the lung (in the spontaneous metastasis model) or liver (in the peritoneal metastasis model) was reported as micrometastases (see Figure 2).

**Table 1:** Metastatic propensity of tumor progenitor (TP) cells isolated from tumor tissue, ascites, and blood of cancer patients, demonstrated using the spontaneous metastasis^a^ and the peritoneal metastasis^b^ models

Type	Patient/stage	Epi^+^ cells per mouse	TP cells per mouse	Frequency
1°‐tumor^a^	P01/III	500 000	300	1/10
	P02/IV	500 000	400	2/11
m‐tumor^a^	P01/III	500 000	300	0/3
	P02/IV	500 000	400	0/3
Ascites^b^	P03/III	50 000	2000	20/23
	P04/IV	50 000	2000	22/23
Blood^a^	P05;P06/Benign	2000	0	0/4
	P07;P08/I	2000	0	0/4
	P09/II	2000	300	0/2
	P10;P11;P12/III	2000	500	1/3
	P13;P14/IV	2000	500	1/2
	P15/IV^c^	2000	2000	2/2

Abbreviations: 1°‐tumor, primary tumor; m‐tumor, metastatic tumor.

a TP cells isolated from tumor tissue and blood were tested in the spontaneous metastasis model.

b TP isolated from ascites were tested in the peritoneal metastasis model.

c iCTCs isolated from a patient with stage IV pancreatic cancer that were amplified in the stem cell media.

To detect iCTCs in mice, individual blood sample was transferred into a CAM‐coated tube (Vita‐Cap™, Vitatex Inc, Stony Brook, NY) and incubated for 3 hours at 37°C. iCTCs and lung micrometastases were examined by fluorescence microscopy and measured for GFP content by immuno‐western blotting. The latter was performed following a procedure previously described^22^; immunoblotting densitometry was performed using ImageQuant TL software (GE healthcare, Piscataway, NJ).

### 2.4 Ascites, peritoneal metastasis models, and GFP labeling to track spreading of tumor cells

Ascites were first spun down at 800 g for 5 minutes to obtain the cellular component. Large tissue and clot fragments were removed with a 150‐µm Nylon screen mesh. To evaluate cell viability, 1 × 10^4^ cells from ascites samples were set aside and incubated with a mixture of 1‐mmol/L ethidium homodimer‐1 (EthD‐1) and 5‐mmol/L Calcein AM (Invitrogen). To estimate numbers of Epi^+^ bulk tumor cells obtained, 1 × 10^5 ^cells were fixed, blocked, and then stained with a mixture (Epi) of FITC‐conjugated primary antibodies against ESA/CD24 (clone VU‐1D9, Biomeda, Foster City, CA) and EPCAM (clone Ber‐Ep4, Dako, Carpinteria, CA), followed by 10 minutes staining with 10‐µg/mL Hoechst 33342 (Invitrogen) and Epi^+^Hoechst^+^ cells were measured under a Nikon TE300 fluorescence microscope (Nikon, Japan). To develop the peritoneal tumor growth and metastasis model, 0.1 to 1 × 10^7^ viable Epi^+^ cells from each sample were re‐suspended in DMEM with 15% FBS and injected *ip* to a 4‐6‐week‐old NOD‐SCID mouse (Jackson Labs, Bar Harbor, Maine). Established xenografts were maintained for multiple passages by *ip* injection of ~5 × 10^6^ ascites tumor cells into a new mouse at each passage.

The GFP‐containing transfer vector plasmid (pRRL‐CMV‐GFP), the helper plasmids (pMDLg/pRRE, pRSV.Rev, and pMD.G), and the packaging cell line (293T) were provided by Dr Scott Lowe from Cold Spring Harbor Laboratory. All plasmids were amplified by transformation into competent *Escherichia coli* and purified. Lentiviruses were produced by transient transfection of the transfer vector plasmid and the helper plasmids into 293T cells. Epi^+^ tumor cells were infected with the GFP‐encoded lentivirus. Approximately 2 × 10^5^ tumor cells were briefly treated with 0.05% Trypsin/EDTA to enhance accessibility of the viruses to cells. The cells were washed with DMEM and infected with the lentivirus for two cycles. At each cycle, the cells were suspended in 2‐mL of medium containing 1.5 × 10^7^ viral particles (MOI = 75) and 8‐µg/mL of polybrene. They were then seeded to a 96‐well plate with 100‐µL per well. The plate was spun at 1800 g for 45 minutes at room temperature and then incubated at 37°C for 3 hours.

### 2.5 General biochemical assays and selection of seprase‐binding peptides

This study utilized techniques such as analyses of protein expression by Western immunoblotting, RNA interference and overexpression plasmid constructs, transfection, and cell cloning. All of these procedures were performed, as described.^21^, ^23^, ^24^

To generate peptide inhibitors against seprase, active seprase, which was isolated from LOX cells, was used as bait for screening peptide inhibitors from phage display peptide libraries. After four rounds of biopanning and six rounds of preadsorption, pronounced enrichment in phage binding to purified seprase was obtained. The consensus nucleotide sequences built from the positive clones defined two potential promising peptides, DMWERVSR and DLDYLSKF. We then synthesized CDMWERVSRC, a cyclic form of DMWERVSR with a previously described method 25 that was, at 100 μmol/L (IC_50_ of 10 μmol/L), 40% more active than its linear counterparts. CDMWERVSRC was also a strong inhibitor against the gelatinase activity of purified seprase (IC_50_ < 10 μmol/L). In contrast, DPP inhibitors including H‐Ile‐Pro‐NHO‐PNB and H‐Ile‐Thia did not effectively inhibit the gelatinase activity of seprase, suggesting that the cyclic CDMWERVSRC peptide is a specific and effective inhibitor that blocks the gelatinase activity of seprase. In vitro, the inhibition of seprase activity in LOX cells by cyclic peptide CDMWERVSRC did not alter the cell proliferation, as assessed using soft agar assay. In addition, CDMWERVSRC, at doses ranging from 10^−4^ to 10^3^ μmol/L, had no effect on cell viability. CDMWERVSRC strongly blocked CAM uptake by LOX cells; however, vehicle medium and control peptide REMSDWRV did not. Similarly, CDMWERVSRC and the MMP inhibitor CT‐1746 reduced the collagen degradation by LOX cells in a dose dependent manner with an IC_50_ of ~200 μmol/L, whereas control peptides, GRGTWN and REMSDWRV, did not. These data show that CDMWERVSRC exhibits low cellular toxicity and inhibits the gelatinase activity of seprase to the extent that it completely abrogates seprase function in cell invasion toward the extracellular matrix.

### 2.6 Cell transfection and establishment of tumor cell lines with contracting levels of seprase expression

Parental cells were single‐cell cloned by limiting dilution and expanded in the medium conditioned by parental LOX and SK‐MEL‐28 cells. New cell sublines were created via LIPOFECTAMINE Plus (GIBCO‐BRL) transfection of pGUS and pGUS‐sep vectors without linearization. Stable transfected sublines were selected using the complete cancer cell (CCC) medium supplemented with G418 (600 μg/mL). G418‐resistant cell sublines were then single‐cell cloned. LOX and SK‐MEL‐28 cells were tagged with the green fluorescent protein (GFP) by transfecting with pCEFG vector, and stable clones were selected in the presence of 400‐μg/mL of G418 (Sigma).

To examine whether the expression of the iCTC marker seprase is involved in metastatic progression, we first established stable cells from the nonseprase‐expressing, nontumorigenic, and nonmetastatic SK‐MEL‐28 (SK) human melanoma line 26 that overexpressed seprase, and that knocked down the induced seprase expression in the progeny cells. SK/OV cells were established by cloning parental SK cells transfected with pGUS vector carrying both GFP and seprase expression inducer c‐Ski,^27^ which eventually forced the overexpression of seprase. Derived from SK/OV cells, SK/OV/SEP‐KD cells were established by introducing a shRNA vector pSEP‐1 that knocked down seprase overexpression. Both SK/OV and SK/OV/SEP‐KD cells were established successfully that mRNA expression, protein expression, gelatinase activity specific for *seprase* gene activity were altered accordingly, as compared with that of the seprase‐expressing LOX cells.

To establish LOX cells with altered seprase expression, we developed a RNAi vector, pGUS, which harbors an enhanced GFP expression cassette and a human U6 promoter sequence.^28^ We transfected the shRNA expression vectors into LOX cells that constitutively express seprase for establishment of cells with seprase knock‐downs. In addition, parental (nonfluorescent) LOX cells transfected with the pGUS vector (GUS‐1 and GUS‐2 seprase^high^ cells) were GFP‐tagged. SEP‐1 seprase^low^ cells had more than 95% of *seprase* mRNA suppressed as measured with qRT‐PCR, while SEP‐2 seprase^low^ cells showed a reduction greater than 80%. Western immunoblotting analysis showed no detectable amount of seprase protein in SEP‐1 and SEP‐2 cells. Consistently, proteolytic assays of immuno‐captured protein revealed that SEP‐1 and SEP‐2 seprase^low^ cells had reduced gelatinase and peptidase activities when compared with GUS‐1 seprase^high^ cells. In vitro*,* a reduction in seprase expression in the SEP‐1 and SEP‐2 cell lines corresponded with a significant (*P* < 0.001) inhibition of these cells to exhibit CAM uptake and degradation.

## 3 RESULTS

### 3.1 Characterization of TP cells in vitro

**Figure 1:**
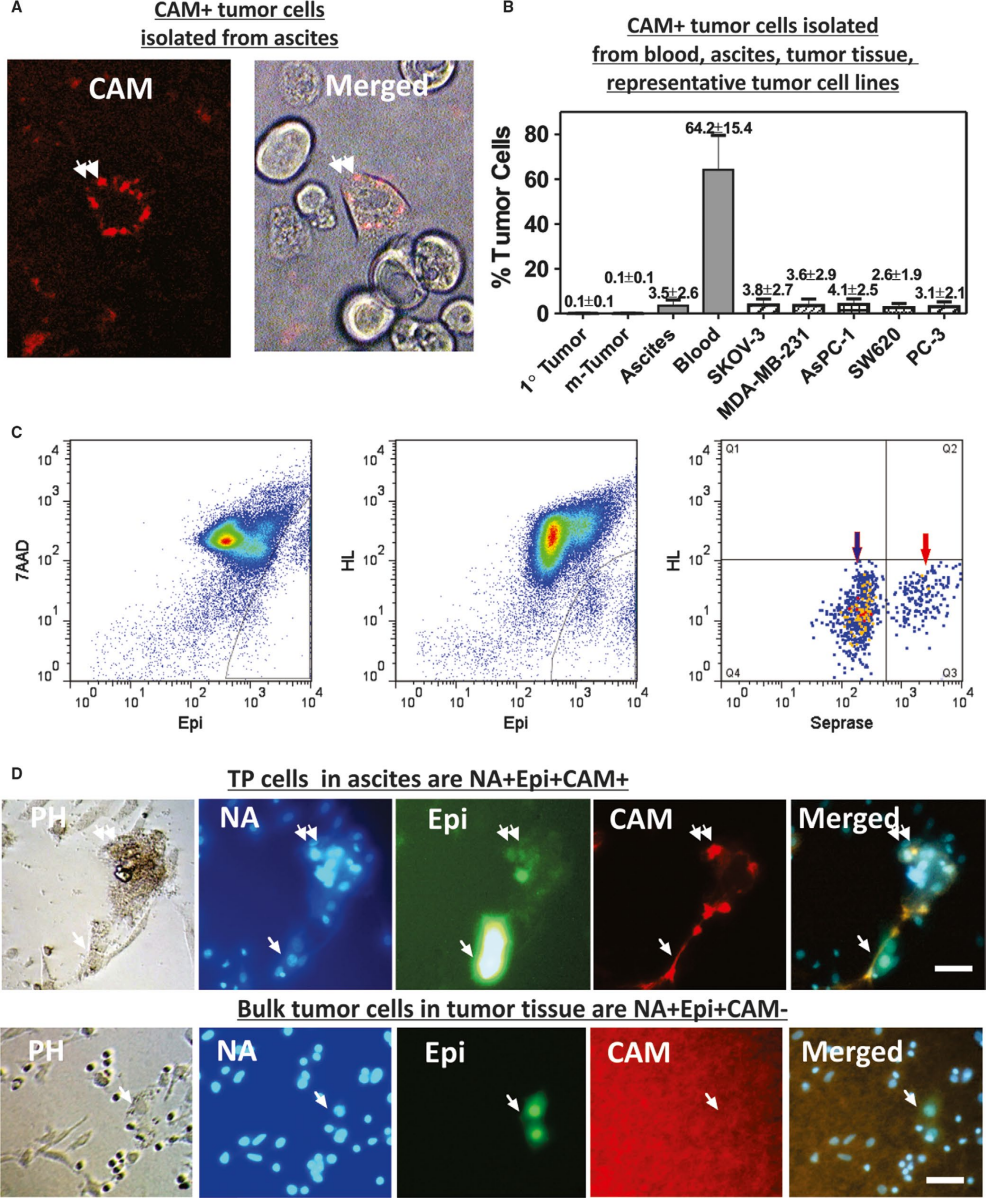
Identification of tumor progenitor (TP) and bulk tumor cells in tumor cell lines and patient‐derived tumor tissue, ascites, and blood. (A) A live TP cell captured by collagen‐based adhesion matrix (CAM) from ascites of an ovarian cancer patient is shown in the direct merge image of phase contrast light and red fluorescence (merged) that exhibits CAM uptake (CAM^+^). (B) Percentage of CAM^+^ TP cells among Epi^+^ bulk tumor cells were estimated in various cellular sources from ovarian cancer patients and established human tumor cell lines listed. (C) Segregation of TP cells (red arrow) from bulk tumor cells (blue arrow) in the blood model spiked with MDA‐MB‐231 cells using HL, Epi and TP marker seprase by flow cytometry. 7AAD is a dye staining cellular nuclei. HL represents hematopoietic lineage cells stained with antibody against CD45. (D) CAM‐avid cells isolated from ascites (upper panel) and primary tumor (lower panel) of an ovarian cancer patient were cultured on the red fluorescently labeled CAM scaffold for 5 days. Cells were fixed with 1% paraformaldehyde, stained with blue‐fluorescent Hoechst 33258 dye (labeled NA), and photographed under phase contrast and fluorescent microscopy. Upper panel: NA^+^Epi^+^CAM^+^ TP cells (double arrows) were depicted as a cluster of 16 cells that degraded/removed the underlying CAM. Lower panel: NA^+^Epi^+^CAM^‐^ bulk tumor cells (arrows) were large cell doublets that did not display CAM uptake nor degradation of underlying CAM. Bar = 50‐µm. The red background is the red fluorescent labeled CAM film underlying the bulk tumor cells (not background fluorescence of bulk tumor) that remain “intact” highly fluorescently due to the lack of fluorescent labeled CAM degradation by the tumor cells. Only the TP cells can effectively degrade the CAM film and the bulk tumor cells cannot

Figure 1 shows the characterization of TP and bulk tumor cells in tumor cell lines and in patient‐derived tumor tissue, ascites, and blood. Bulk tumor cells were defined by the Epi^+^ cells expressing epithelial lineage (Epi) markers EPCAM and CD24. TP cells were identified to be exhibiting functional CAM uptake (CAM^+^, Figure 1A,D), co‐expression of Epi^+^ and invasion/stem (TP) markers seprase/CD44 (NA^+^Epi^+^TP^+^, Figure 1C), or co‐expression of Epi^+^ and CAM^+^ (NA^+^Epi^+^CAM^+^, Figure 1D). TP cells were found in less than 0.1% of the Epi^+^ bulk tumor cells in primary and metastatic tumors (n = 19) and 3.5 ± 2.6% of the Epi^+^ bulk tumor cells in ascites (n = 20) from EOC patients (Figure 1B). Although the presence of immune and stromal cells in tumors and in ascites is expectedly different, the result showed % of TP cells against Epi^+^ bulk tumor cells in different specimens and had no inference on the presence of immune and stromal cells in tumors and ascites.

Frequencies of TP cells in blood, iCTCs, were the highest among various cellular sources, that is, 64.2%±15.4% of Epi^+^ cells in blood (n = 49 ovarian cancer).

Human tumor cell lines, which were obtained from the ATCC and derived from the body fluid of cancer patients, included ovarian tumor line SKOV‐3, breast tumor cell MDA‐MB‐231, pancreatic tumor cell AsPC‐1, colon tumor cell SW620, and prostate tumor cell PC‐3, were spiked into healthy blood and assayed on CAM‐coated plates to assess the presence of NA^+^Epi^+^CAM^+^ TP cells (Figure 1B). We found that these cell lines contained 2%‐4% TP cells, frequencies consistent with those of patient's ascitic fluids, in which cell lines were derived. We also found that the highly metastatic human melanoma line LOX contained high % of TP cells (24.8%±15.9%, n = 20), suggesting that invasiveness of LOX cell lines assessed by the CAM uptake assay in vitro could be consistent with the metastatic propensity of the cell lines in vivo (see below).

Growth and morphology of TP and bulk tumor cells from ascites and solid tumors of EOC patients were examined using cells captured on a plate coated with CAM and continuously cultured in a standard medium containing 10% serum for 5 days (Figure 1D for an example). TP cells, but not bulk tumor cells, locally degraded the underlying CAM film and ingested the red fluorescent CAM material (Figure 1D, double arrows). TP and bulk tumor cells formed colonies in culture, 98 out of 135 samples tested (72.6%); the rest were proliferating as solitary cells. At day‐5, colonies composed of 16‐32 TP cells were found (Figure 1D, upper panel), suggesting a doubling time of about 34‐42 hours for TP cells. However, at day‐5, colonies composed of two bulk tumor cells from solid tumors were found (Figure 1D, lower panel), suggesting a doubling time of about 120 hours for bulk tumor cells.

### 3.2 Proliferation and metastasis of TP cells in vivo

**Figure 2:**
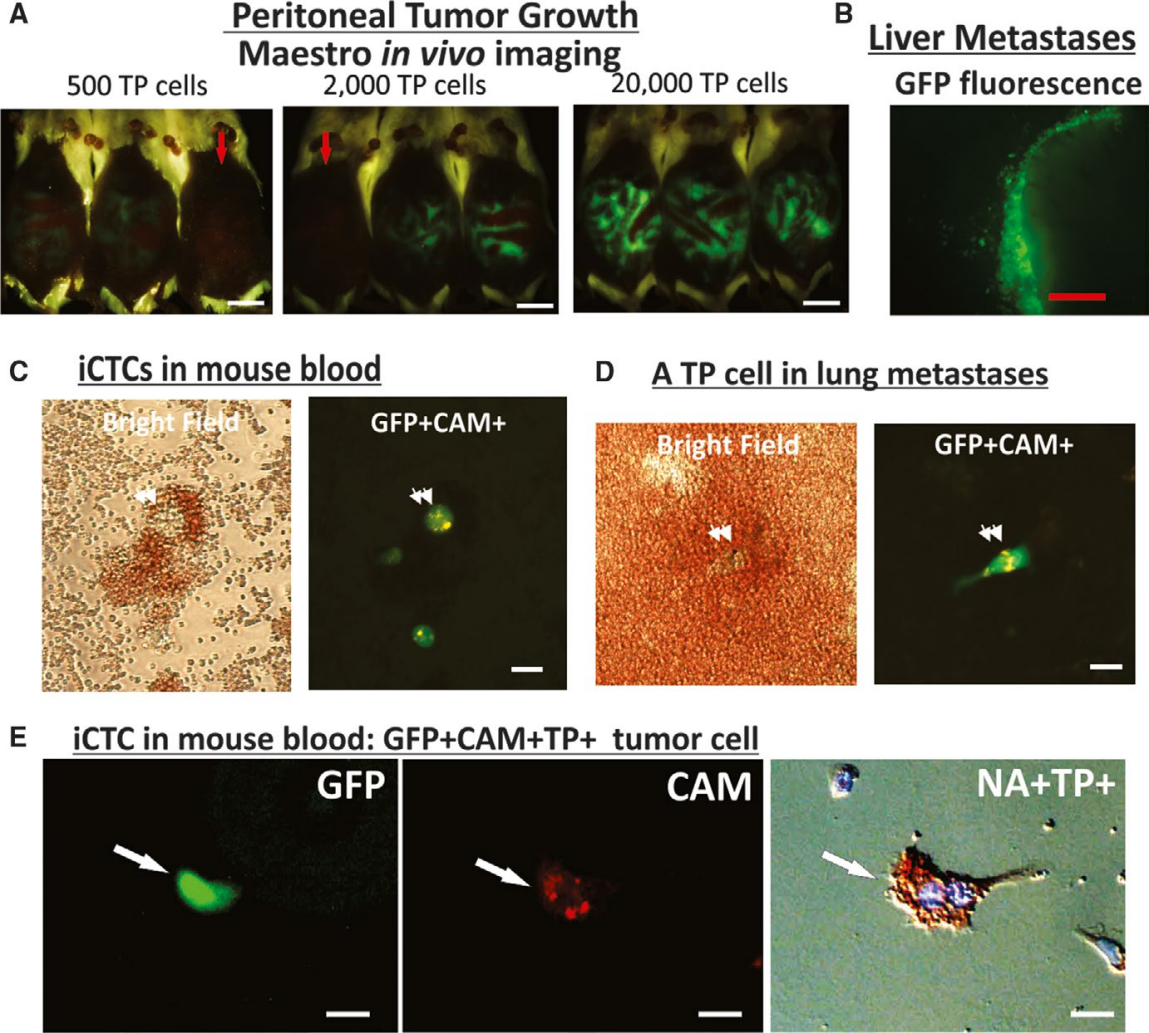
GFP‐tagging of collagen‐based adhesion matrix (CAM)‐avid tumor cells from patients to verify the proliferative and metastatic properties of tumor progenitor (TP) cells in vivo. (A) Peritoneal tumor growth of inoculated GFP‐tagged tumor cells derived from ascites of an ovarian cancer patient. Using the peritoneal metastasis mouse model, 500, 2000, and 20 000 TP cells, respectively, were injected *ip*. Growth of GFP‐tumor cells in the peritoneal cavity was revealed by Maestro system. Mouse peritoneal cavities that contained GFP‐tagged tumor cells were seen in the experimental mice but not in the control (red arrows in 500 and 2000 TP cells panels). Bar = 1‐cm. (B) Liver micrometastases of a peritoneal metastasis model were revealed by fluorescence microscopy and seen as clusters on the surface of the dissected organ. Bar = 1‐mm. (C) iCTCs detected in mouse blood of a spontaneous metastasis model were seen in GFP^+^CAM^+^ merged image (double arrows). Bar = 20‐µm. (D) A TP cell isolated from mouse lung of the spontaneous metastasis model was seen in GFP^+^CAM^+^ merged image (double arrows). Bar = 20‐µm. (E) An iCTC detected in mouse blood of the spontaneous metastasis model was GFP^+^CAM^+^NA^+^TP^+^. Bar = 20‐µm

The capability of TP cells to proliferate and metastasize in vivo was verified using GFP tagging of CAM‐avid tumor cells derived from ascites of cancer patients in a mouse peritoneal metastasis model (Figure 2A‐E). For examples, tumor cells captured by CAM from ascites of a patient with stage III ovarian cancer were found to grow in the peritoneal cavity (Figure 2A) and formed liver micrometastases (Figure 2B). Mice inoculated with 500, 2,000, and 20,000 CAM‐avid TP cells produced tumors with increasing GFP‐signal in the peritoneal cavity as revealed by the Maestro imaging system (Figure 2A), and GFP‐tumor cells that metastasized to the liver were observed (Figure 2B). Tumor cells emigrated in mouse blood (Figure 2C) and seeded in the lung (Figure 2D) were in turn captured and identified using the CAM uptake Vita‐Assay™ invasion assay, showing iCTCs and liver or lung micrometastases that retained the TP phenotype as GFP^+^CAM^+^; and expressed seprase as well (Figure 2E).

To assess metastatic propensity of TP cells derived from primary and metastatic tumor tissues and blood of patients, serial numbers of CAM‐avid cells were inoculated *sc* into SCID mice to establish the spontaneous metastasis model. In both peritoneal and spontaneous metastasis models, a minimal of 500 TP cells could generate iCTCs and micrometastases in mice (Table 1), a degree of metastatic spread equivalent to 2 × 10^5^ Epi^+^ tumor cells isolated from primary and metastatic tumors and ascites (Table 1). However, palpable tumors were only found in mice when 2 × 10^5^ bulk tumor cells were injected. The results showed that TP cells derived from patient's specimens recapitulated their invasiveness using the in vitro CAM uptake assay and their metastatic potential using in vivo metastasis models.

### 3.3 Seprase expression drives formation of iCTCs and micrometastases

**Figure 3:**
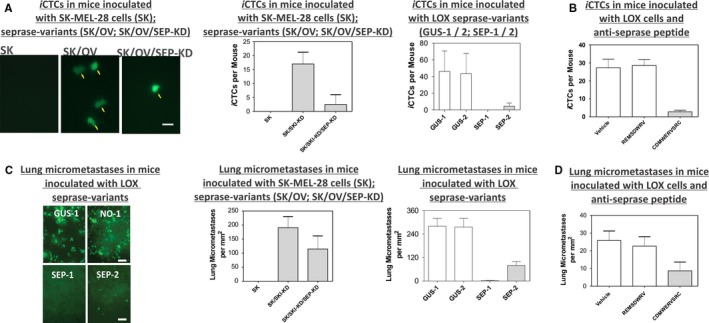
Inhibition of iCTCs in blood and micrometastases at the lung in the spontaneous metastasis model using alternation of seprase gene expression and anticatalytic seprase peptides. Blood and lung specimens were extracted by cardiac puncture of mice at the term. (A) Epifluorescence microscopic identification and measurement of iCTCs in SCID mice that were inoculated with SK‐MEL‐28 cells (SK) that express no detectable seprase, SK cells that overexpress seprase (SK/OV) and SK/OV cells that were knock‐down seprase expression with seprase RNAi (SK/OV/SEP‐KD), as well as LOX cell variants that express high levels of seprase (GUS‐1 and GUS‐2) or had knock‐down seprase expression using RNAi (SEP‐1 and SEP‐2). In image, Bar = 10‐μm. In plots, each data point represents mean ± SEM (n = 5). *P* < 0.01, *t* test. (B) suppression of metastatic spread of seprase‐expressing LOX tumor cells into the blood as iCTCs by the inhibitory peptide CDMWERVSRC against seprase. SCID mice were co‐inoculated with GFP‐LOX cells and CDMWERVSRC, REMSDWRV or control vehicle DMEM medium. (C) Epifluorescence microscopic identification and measurement of lung micrometastases in SCID mice that were inoculated with SK‐MEL‐28 cells (SK) that express no detectable seprase, SK cells that overexpress seprase (SK/OV) and SK/OV cells that were knock‐down seprase expression with seprase RNAi (SK/OV/SEP‐KD), as well as LOX cell variants that express high levels of seprase (GUS‐1 and GUS‐2) or had knock‐down seprase expression using RNAi (SEP‐1 and SEP‐2). (D) suppression of metastatic spread of seprase‐expressing LOX tumor cells to the lung as micrometastases by the inhibitory peptide CDMWERVSRC against seprase. SCID mice were co‐inoculated with GFP‐LOX cells and CDMWERVSRC, REMSDWRV or control vehicle DMEM medium

To examine if a tumor cell with altered seprase expression can spread to blood as iCTCs, and to the lung as micrometastases, we inoculated GFP‐labeled tumor cells *sc* and examined GFP‐labeled iCTCs in blood (Figure 3A,B) and micrometastases in the lung (Figure 3C,D). The induced expression of seprase in SK/OV cells caused a significant increase in iCTCs (Figure 3A, *P* < 0.001) and lung micrometastases (Figure 3C, *P* < 0.001). The subsequent knockdown expression of seprase in SK/OV/SEP‐KD cells in turn generated significantly less iCTCs (Figure 3A, *P* < 0.001) and lung micrometastases (Figure 3C, *P* < 0.001) than these from SK/OV cells.

To determine if cells with suppressed seprase expression alter their metastatic propensity, GFP‐tagged LOX cells with contrasting levels of seprase expression were used to test in the spontaneous metastasis model (Figure 3A,C). Mice were *sc* inoculated, 6 × 10^5^ cells/mouse, with each cell line, including seprase^high^ GFP LOX cells (GUS‐1) and seprase^low^ GFP LOX cells (SEP‐1 or SEP‐2). GFP tagged iCTCs and micrometastases were examined in mice 14 days after tumor cell inoculation. While seprase^high^ GFP LOX cells (GUS‐1 or GUS‐2) generated significant numbers of iCTCs in blood and micrometastases in the lung, suppression of seprase expression in seprase^low^ GFP LOX cells (SEP‐1 or SEP‐2) produced primary tumors that could not spread or spread to less extent as iCTCs and lung micrometastases (Figure 3A,C). The above seprase‐expression experiments show that expression of seprase in transfected tumor cells induces metastatic spread of these cells in blood as iCTCs. Convertibly, lack of seprase expression and knock‐down seprase expression suppresses metastatic spread of tumor cells into the blood.

### 3.4 Perturbations of seprase functionality alter formation of iCTCs and micrometastases

To examine the role of the seprase activity in metastasis, mice were co‐injected *sc* with GFP‐LOX cells, 2 x 10^5^ cells/mouse, and CDMWERVSRC inhibitor (the experimental group), control peptide REMSDWRV or control vehicle (the control groups), followed by daily injections of specific peptides. After 14 days, weights of tumors developed in the primary sites were measured that showed no significant difference among tumors treated with CDMWERVSRC, REMSDWRV, and control vehicle, affirming that seprase activity does not affect proliferation of inoculated human cells and subsequent tumor growth. Importantly, the antiseprase peptide inhibitor CDMWERVSRC reduced significantly the numbers of iCTCs and lung micrometastases, whereas REMSDWRV and control vehicle did not (Figure 3B,D, *P* < 0.05).

**Figure 4:**
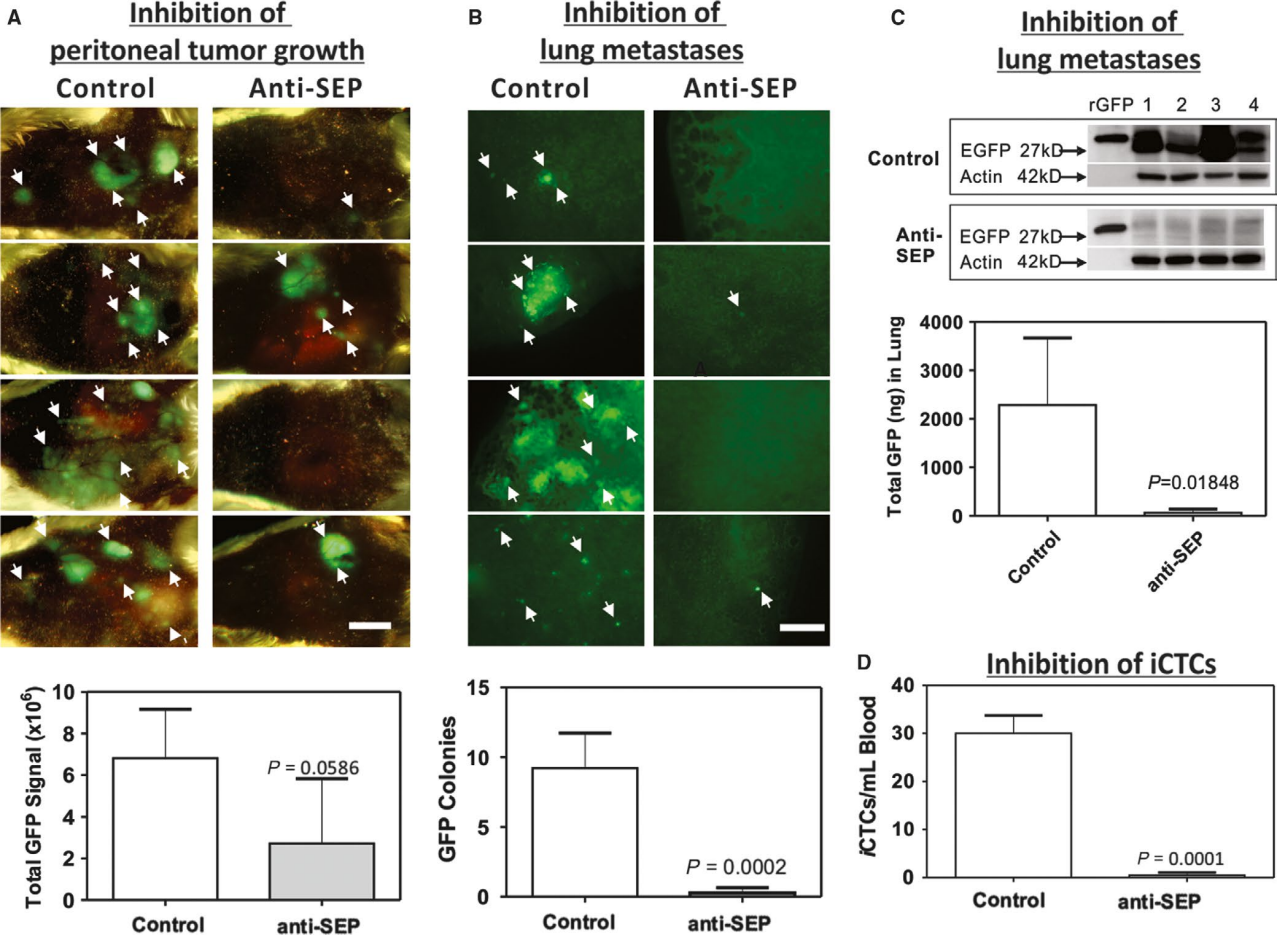
Inhibition of iCTCs and lung micrometastases using antibodies against seprase (SEP). In plots, each data point represents mean ± SEM (n = 5). *P* < 0.01, *t* test. (A) Maestro imaging of GFP‐tumor growth in the peritoneal cavity of experimental (treated with anti‐SEP antibodies) and control mice. Note that peritoneal tumor growth was not significantly blocked by anti‐SEP antibodies. Bar = 1‐cm. (B) Inhibition of lung metastases by anti‐SEP antibodies as seen with the presence of GFP colonies. Bar = 40‐µm. (C) Inhibition of lung micrometastases by anti‐SEP antibodies as shown in Western immunoblotting of lung extracts from experimental and control mice. (D) Inhibition of iCTCs in mouse blood as seen with the presence of GFP‐tagged CTC colonies in blood when mice were treated with anti‐SEP antibodies

Subsequently, we used the peritoneal metastasis model to examine whether iCTCs and liver micrometastases could be blocked by inhibitory antibodies against seprase on the surface of TP cells (Figure 4). SCID mice were *ip* co‐inoculated with 1 × 10^5^ GFP‐tagged MDA‐MB‐231 tumor cells and antiseprase D28/E19 antibodies, followed by weekly *ip* application of antibody solution for 42 days. Since the peritoneal cavity is the tumor cell inoculation site, we used Maestro^TM^ in vivo imaging and overall GFP signal strength in a plot to reveal the extent of tumor growth and/or peritoneal metastasis at term of the experiment (Figure 4A).

To examine the effect of inhibitory antiseprase antibodies on metastasis, micrometastases in the lung were measured in dissected organs by quantifying GFP using fluorescence microscopy and Western immunoblotting (Figure 4B,C). We found that micrometastases in the lung were significantly reduced in the experimental group as compared to the control group (Figure 4B, *P* < 0.0002;4c, *P* < 0.02). Similarly, iCTCs in the mouse blood were significantly reduced in the experimental group as compared to the control group (Figure 4D, *P* = 0.0001). These data demonstrate a role of active seprase on the TP cell in metastasis.

## 4 DISCUSSION

Recent advances in CTC research have shown that CTCs are important clinical indicators for predicting metastatic progression and treatment efficacy.^8^, ^9^, ^10^, ^19^ However, CTC investigation is hampered by their low number and heterogeneity. Heterogeneity is commonly observed among various specimens from cancer patients including that of CTCs. Although CTCs provide a source of readily available and sequentially sampled invasive cancer cells suitable for developing ex vivo cultures of patient‐specific tumor models for the individualized therapeutic evaluation of cancers,^4^, ^6^, ^7^ a specific population of CTCs that have the metastatic proclivity have not been well‐investigated. We report here the isolation and characterization of the invasive population of CTCs, iCTCs, to establish a novel patient‐derived CTC model that recapitulated the metastatic process by producing iCTCs in the blood and micrometastases in the lung of mice and have replicated the treatment responsiveness using therapies against seprase, an iCTC marker. We showed that tumor cells isolated by the functional CAM method from ascites and solid tumors of EOC patients exhibited TP and bulk tumor cell populations in vitro: CAM^+^ or TP^+^ TP cells and Epi^+^ bulk tumor cells. Our observation that tumor cells emerging in blood and bodily fluid contained high frequencies of TP cells is consistent with the canonical hypothesis of metastatic progression that CTCs isolated using various cell enrichment methods contain a significant population of metastasis‐initiating tumor cells,^4^, ^5^ the iCTC population we describe in the present study.

We showed that, in both peritoneal and spontaneous metastasis models, a minimal of 500 TP cells could recapitulate invasive and metastatic phenotypes of the TP cell and generate iCTCs and micrometastases in mice, a degree of metastatic spread equivalent to 2 × 10^5^ Epi^+^ tumor cells isolated from primary and metastatic tumors and ascites from patients or to that of one million tumor cells in classical metastasis (cell line) studies. We showed the metastatic propensities of patient‐derived TP cells that could spread to blood as iCTCs and to the lung and liver as micrometastases in peritoneal and spontaneous metastasis models. In this respect, TP cells meet in part a commonly accepted criterion for cancer stem cells 29, 30 or metastasis‐initiating cells 4, 5 because they consistently produced iCTCs and micrometastases when introduced into experimental animals.

To gain some insights on cancer progenitor‐like properties and the metastatic potential of iCTCs, 2000 iCTCs that were isolated from blood of a pancreatic cancer patient and amplified by culturing for 2 weeks in a stem cell medium were used to inoculate one individual mice in comparison with 0‐500 iCTCs freshly isolated from blood samples of 10 ovarian patients (Table 1). We were reasoning that serial numbers of iCTCs (0, 25, 100, 500, 2000 etc.) isolated from individual patients regardless their cancer types, that is, ovarian and pancreatic cancers, that shared the Epi^+^TP^+^HL^‐^ phenotype were available at time of in vivo testing (Table 1). For examples, there were only less than a few thousands of iCTCs freshly isolated from the 10 individual ovarian patients, but more than 200,000 iCTCs that were freshly isolated by CAM from a pancreatic patient and amplified by culturing cells in a stem cell medium for 2 weeks were available. The result showed that more than 500 iCTCs with the identical Epi^+^TP^+^HL^‐^ phenotype that were derived from ovarian and pancreatic cancer patients could exhibit cancer progenitor‐like properties and metastatic propensity in mice. However, this study provides no evidence to discriminate the metastatic potential of iCTCs derived from pancreatic and ovarian cancer patients.

In this paper, we investigated the role of iCTCs in assessing therapeutic responsiveness using gene expression and proteolytic activity inhibitory approaches targeting the TP marker seprase. Seprase was chosen as the therapeutic target because it was expressed on the surface of iCTCs.^21^, ^23^ Seprase and DPP4 assemble into a complex on the surface of carcinoma cells that may convert Met‐α2‐antiplasmin (AP) to the fibrin‐incorporable form, Asn‐α2‐AP, that, in turn, increase the fibrin—AP microclot surrounding certain tumor cells and may impact tumor cell viability and therapeutic susceptibility.^21^, ^31^ DPP4, a dipeptidyl prolyl peptidase homologue of seprase, is constitutively expressed in epithelial and carcinoma cells that assembles with seprase on the cell surface.^32^ Our seprase‐expression experiments show that metastasis, as measured by formation of iCTCs and lung micrometastases in experimental mice, depends on the presence of seprase‐expressing cells, that is, TP cells, in a tumor. Seprase‐inhibition experiments demonstrated that functional seprase on the surface of TP cells promotes their intravasation into the bloodstream as iCTCs and formation of micrometastases in the lung. Consistent with our conclusion on the therapeutic potential of seprase inhibition, other works have recently demonstrated the role of seprase/FAP inhibition in immunotherapy.^33^, ^34^, ^35^ A full preclinical study to demonstrate survival benefit is therefore warranted to address the therapeutic potential of seprase or iCTCs as a whole in the near future.

In summary, this study demonstrated that iCTCs isolated by the CAM method from blood of EOC patients had 100‐fold greater invasive and metastatic potentials than bulk tumor cells from other tissues. We showed that the invasiveness of a TP cell assessed by the CAM uptake assay in vitro directly related with its metastatic potential in vivo and that patient‐derived iCTCs could metastasize to mouse blood as iCTCs and to the lung and liver as micrometastases in peritoneal and spontaneous metastasis models. In addition, we validated the role of iCTCs in assessing the therapeutic responsiveness using the reduced formation of iCTCs and micrometastases in two metastasis models by RNAi, peptides, and monoclonal antibodies against the iCTC marker seprase.

## AUTHOR CONTRIBUTIONS

HD, MLP, and WC, had the initial idea of this project, wrote the manuscript, and conceived the project and supervised all research. HD, ST, QZ, LC, and DC performed the majority of experiments, prepared most data presentations and wrote the manuscript. MLP provided and analyzed clinical data.
